# Diagnostics in Patients Suspect for Breast Cancer in The Netherlands

**DOI:** 10.3390/curroncol28060419

**Published:** 2021-11-29

**Authors:** Madelon M. Voets, Catharina G. M. Groothuis-Oudshoorn, Liset H. J. Veneklaas, Srirang Manohar, Mariël Brinkhuis, Jeroen Veltman, Linda de Munck, Lioe-Fee de Geus-Oei, Mireille J. M. Broeders, Sabine Siesling

**Affiliations:** 1 Department of Health Technology and Services Research, Technical Medical Centre, University of Twente, P.O. Box 217, 7500 AE Enschede, The Netherlands; m.m.voets@utwente.nl (M.M.V.); c.g.m.oudshoorn@utwente.nl (C.G.M.G.-O.); lisetveneklaas@gmail.com (L.H.J.V.); 2 Multi-Modality Imaging, Technical Medical Centre, University of Twente, P.O. Box 217, 7500 AE Enschede, The Netherlands; s.manohar@utwente.nl; 3 Laboratory for Pathology East Netherlands, LabPON, Boerhaavelaan 59, P.O. Box 516, 7550 AM Hengelo, The Netherlands; m.brinkhuis@labpon.nl; 4 Department of Radiology, Ziekenhuisgroep Twente, Zilvermeeuw 1, P.O. Box 7600, 7609 PP Almelo, The Netherlands; j.veltman@zgt.nl; 5 Department of Research and Development, Netherlands Comprehensive Cancer Organisation, P.O. Box 19079, 3501 DB Utrecht, The Netherlands; l.demunck@iknl.nl; 6 Department of Radiology, Leiden University Medical Center, P.O. Box 9600, 2300 RC Leiden, The Netherlands; L.F.de_Geus-Oei@lumc.nl; 7 Biomedical Photonic Imaging Group, Technical Medical Centre, University of Twente, P.O. Box 217, 7500 AE Enschede, The Netherlands; 8 Dutch Expert Centre for Screening, P.O. Box 6873, 6503 GJ Nijmegen, The Netherlands; Mireille.Broeders@radboudumc.nl; 9 Department for Health Evidence, Radboud Institute for Health Sciences, Radboud University Medical Center, P.O. Box 9101, 6500 HB Nijmegen, The Netherlands

**Keywords:** diagnostic care pathway, diagnostic activities, breast cancer, benign breast diagnosis

## Abstract

The goal of this study was to describe the variation in hospital-based diagnostic care activities for patients with symptomatology suspect for breast cancer in The Netherlands. Two cohorts were included: the ‘benign’ cohort (30,334 women suspected of, but without breast cancer) and the ‘malignant’ cohort (2236 breast cancer patients). Hospital-based financial data was combined with tumor data (malignant cohort) from The Netherlands Cancer Registry. Patterns within diagnostic pathways were analyzed. Factors influencing the number of visits and number of diagnostic care activities until diagnosis were identified in the malignant cohort with multivariable Cox and Poisson regression models. Compared to patients with benign diagnosis, patients with malignant disease received their diagnosis less frequently in one day, after an equal average number of hospital visits and higher average number of diagnostic activities. Factors increasing the number of diagnostic care activities were the following: lower age and higher cM-and cN-stages. Factors increasing the number of days until (malignant) diagnosis were as follows: higher BIRADS-score, screen-detected and higher cN-and cT-stages. Hospital of diagnosis influenced both number of activities and days to diagnosis. The diagnostic care pathway of patients with malignant disease required more time and diagnostic activities than benign disease and depends on hospital, tumor and patient characteristics.

## 1. Introduction

Breast cancer is the most common cancer diagnosis and a substantial cause of cancer death in women worldwide, accounting for 25% of all cancers and 15% of all cancer deaths in women in 2018 [1]. In The Netherlands, the number of women diagnosed with breast cancer has risen from 8300 in 1990 to 17,000 in 2019 [2,3]. As per the Dutch Breast Cancer Guideline, patients enter the diagnostic pathway for breast cancer when referred by their general practitioner, having complaints or when referred from screening (mammography scores 0, 4 or 5 in the Breast Imaging Reporting and Data System (BI-RADS) [3]. In The Netherlands a national breast cancer screening program was introduced in 1990 inviting women between ages 50 and 75 for a biennial mammography [4].

After referral, several imaging modalities may be used for the hospital-based diagnostic work-up of suspected breast lesions: mammography, digital breast tomosynthesis, conventional or 3D ultrasound (US) imaging, and magnetic resonance imaging (MRI). In most patients, diagnostic mammography is the indicated first choice imaging modality. US examination is used in addition to mammography for the evaluation of palpable lesions or positive mammographic findings. Due to the proven benefit of ultrasound examination compared to mammography in young women (below 30 years of age), ultrasound is the examination of first choice in symptomatic young, pregnant or breastfeeding patients [3,5]. MRI is mainly used in women with elevated risk for breast cancer, for therapy monitoring or to clarify inconclusive diagnostic results [3]. Triple diagnostics, which includes clinical breast examination with mammography, ultrasound and biopsy, remains the mainstay of an accurate breast cancer diagnosis [3]. Same-day diagnosis has proven high diagnostic accuracy, limits the number of hospital visits before diagnosis and can reduce patient anxiety [6].

In The Netherlands, it is unknown whether the recommendations of the Dutch Breast Cancer Guideline on the diagnostic care pathway are implemented, which type and number of diagnostic modalities (total and per hospital visit) are performed in daily practice, and what the number of hospital visits until diagnosis is. Moreover, it is not known which factors influence this diagnostic work-up.

The primary aim of this study is to describe the hospital-based diagnostic care pathway of patients who come in for a diagnostic work-up in The Netherlands and to identify factors which influence the variation of diagnostic activities performed.

## 2. Materials and Methods

Two different diagnostic pathways have been evaluated, one for patients with a benign diagnosis and one for patients with a pathology-confirmed malignant diagnosis. Therefore, two cohorts were constructed: the benign and malignant cohorts. 

### 2.1. Database Sources

Two data sources were used: hospital-based financial registries obtained from Performation and The Netherlands Cancer Registry (NCR). Performation is a company specialized in supporting healthcare institutions with their information regarding the delivered care activities as cataloged in the diagnosis and treatment combinations (DBC) and registered by the hospitals. DBCs provided information about care activities’ delivered date, medical diagnosis and procedures according to the ICD-10 [7]. For the benign cohort, only this hospital financial data was available. For the malignant cohort, additional data of the detected breast cancer was obtained from The Netherlands Cancer Registry (NCR), a population-based cancer registry hosted by The Netherlands Comprehensive Cancer Organisation (IKNL). Data of patient and tumor characteristics, such as age, gender, tumor histology, topography and stage, is registered for all patients diagnosed with a malignancy directly from patient files of all 89 hospitals in The Netherlands, by means of specially trained data managers. Data on diagnostic activities (Performation) were linked to the patient and tumor characteristics (NCR) using pseudonym patient identifiers. 

### 2.2. Study Population

Of 33 Dutch hospitals approached, nine hospitals participated of which six hospitals approved the utilization and linkage of the financial data to the NCR data for the patients with malignant disease. Patients included were female and male, older than eighteen years of age and had their diagnostic care activities and final diagnosis in 2014 or 2015. The benign cohort consisted of 30,334 patients suspect for breast cancer who had one or more diagnostic imaging care activities delivered, yet were not diagnosed with malignant disease. The malignant cohort consisted of 2236 patients with pathologically (invasive or ductal/lobular carcinoma in situ (DCIS/LCIS)) confirmed breast cancer and were registered in the NCR.

### 2.3. Diagnostic Care Activities

Care activities performed in 2014 and 2015 and those that are specific for breast (cancer) diagnostics and imaging were included. It was presumed that patients were suspect of breast cancer from the moment either one of four breast cancer specific diagnostic care activities, i.e., mammography, breast ultrasound, breast MRI and mammoscintigraphy, first occurred. Since the exact moment of benign diagnosis was unknown, all diagnostic activities after negative breast cancer specific diagnostic care activities were included. Patients with malignant disease were assumed to have received a breast cancer diagnosis at the first pathological confirmation or when an imaging result of malignant neoplasm breast was coded in the hospital database. Care activities that were delivered after the initial day of breast cancer diagnosis were presumed to be part of the treatment or follow-up and were therefore not considered as part of the diagnostic care pathway. Information on clinical breast examination was not available; thus, in this study threefold diagnostics was defined as the combination of mammography, ultrasound and biopsy during the same hospital visit.

### 2.4. Statistical Analysis

The diagnostic pathway of both cohorts was described by the type and number of diagnostic care activities in total, per patient and per hospital visit. Diagnostic care activities performed during the same hospital visit were grouped together and the combination of mammography, breast ultrasound (US) and pathology diagnosis means any number of mammography, breast US and pathology diagnosis. The number of hospital visits per patient was determined. Two-sample *t*-tests and chi-squared tests were used to test for significant differences between the cohorts.

We described the variation in the diagnostic care pathway in the benign and malignant cohort, focusing on the first three hospital visits, counting the most frequently used combination of diagnostic care activities, the total number of diagnostic care activities performed, the proportion of final diagnoses and further diagnostic activities if no final diagnosis was made. 

Furthermore, for the malignant cohort only, the time until diagnosis was calculated as the number of actual days between the first diagnostic care activity and the date of diagnosis. Factors associated with the total number of breast cancer specific diagnostic care activities were determined with a Poisson regression model. Factors associated with time (in days) until diagnosis were determined with a Cox regression model. The following NCR variables were included: age at diagnosis (continuous), relation to population screening (no/yes), BIRADS (0/1/unknown, 3/4 and 5), multifocality (no/yes), palpable (yes/no), cT (0 to 4), cN (0 to 3), cM (no/yes), grade (low, intermediate, high, unknown), localization in the breast (inner, outer, central, overlapping), histology (invasive, ductal, lobular, mixed, other), lateralization (left, right), neo-adjuvant treatment (no, yes, no surgery) and hospital (1 to 6). Chi-square tests were used to test for significance of categorical variables. *p*-values of <0.05 were considered to be significant. Stata (StataCorp. 2015. *Stata Statistical Software: Release 14*. College Station, TX, USA: StataCorp LP., College Station, TX, USA) was used for analysis.

### 2.5. Medical Ethical Approval

This study was approved by the medical ethical review board of the NCR. Hospitals authorized Performation to deliver their financial data to IKNL. Data was linked using pseudonyms to guarantee privacy of the patients.

## 3. Results

The benign cohort included 30,334 patients originating from nine different Dutch hospitals and were aged between 18 and 98 years (mean 50.8). The malignant cohort consisted of 2236 patients originating from six different Dutch hospitals and were aged between 19 and 94 years (mean 61.9). The percentage of patients below 40 years is 23% in the benign cohort compared to 5% in the malignant cohort.

**Figure 1:**
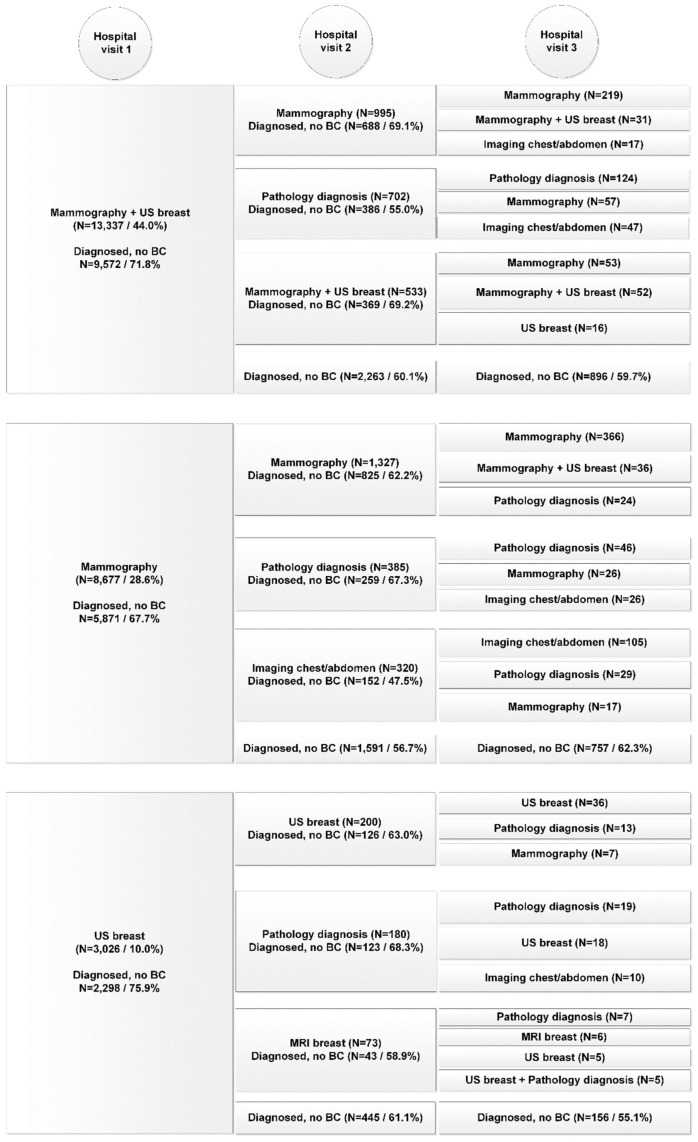
Diagnostic pathway per consecutive hospital visit of patients in the benign cohort (*n* = 30,334).

**Figure 2:**
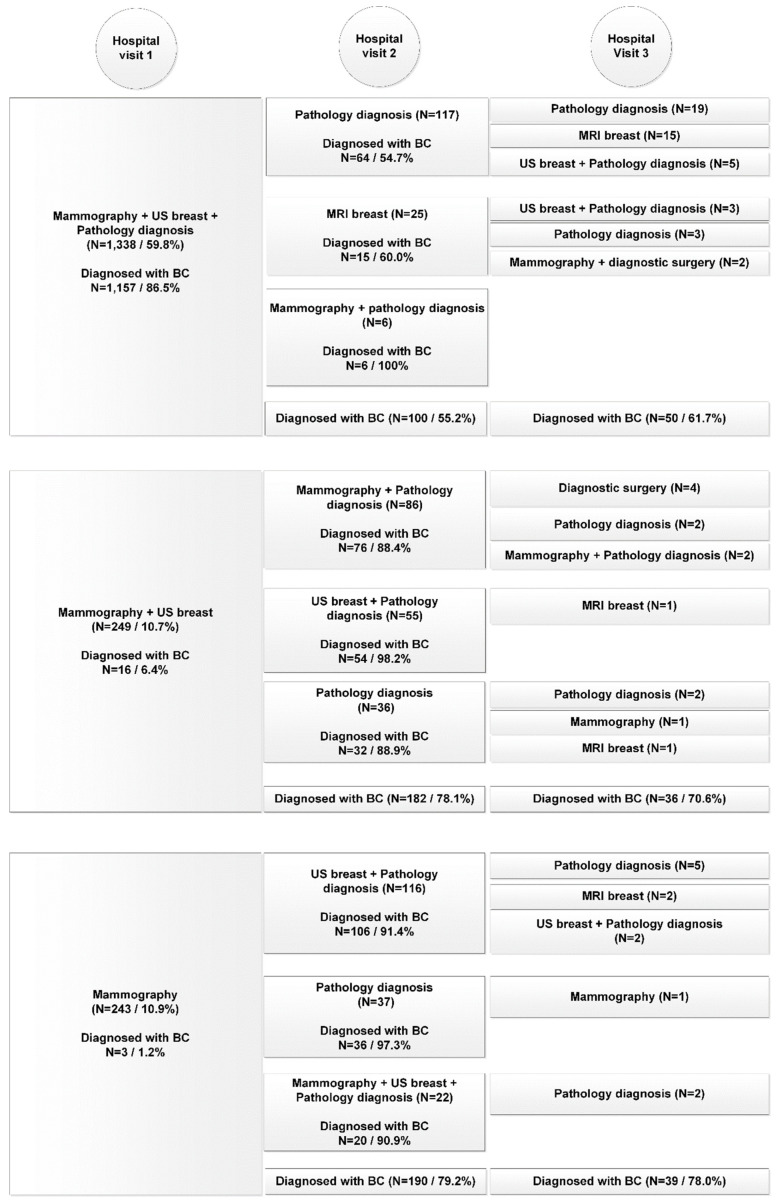
Diagnostic pathway per consecutive hospital visit of patients in the malignant cohort (*n* = 2236).

The variations in diagnostic care pathways for both the benign cohort and malignant cohort are presented for the top-three activities performed, over the course of three consecutive hospital visits in Figure 1 and Figure 2. Horizontally aligned care activities belong to the same diagnostic care pathway. The number of patients diagnosed is the total number of patients diagnosed during the hospital visit of all diagnostic care activities combined. Only the top-three activities performed during each hospital visit for each care pathway are displayed. If less than three activities are presented, no additional diagnostic tests were performed.

In the benign cohort in total 80,153 diagnostic care activities were performed (on average 2.6 per patient, range 1–53) compared to 10,483 (on average 4.7 per patient, range 1–21) in the malignant cohort (*p* < 0.001). The average number of hospital visits was 1.6 for both cohorts (95%-CI (1.60, 1.63)). In the benign cohort 67% had one hospital visit before diagnosis, compared to 62% in the malignant cohort (*p* < 0.001).

In contrast to the benign diagnostic pathway, the malignant diagnostic pathway often included a combination of diagnostic care activities during hospital visits instead of single care activities. Also care activities performed on the first diagnostic care day were less frequently repeated on the second and third care days. In addition, whereas the benign pathway exclusively presented imaging activities or pathology diagnosis, MRI of the breast and diagnostic surgery were introduced in the malignant pathway. 

Patients often underwent more than one diagnostic procedure during their first hospital visit, with 41.3% of the benign cohort and 17.8% of the malignant cohort only getting one care activity delivered during their first hospital visit. 

Of all patients diagnosed during the first hospital visit, 87.1% of patients were diagnosed by one of the top three most common (combination of) diagnostic care activities in the benign cohort and 85.0% in the malignant cohort. In the benign cohort mammography combined with breast US was most frequently performed (44%) resulting in a final diagnosis of no breast cancer in 71.8%. In the malignant cohort 10.7% of the patients (*n* = 249) received only these two diagnostics the first day, which resulted in a final diagnosis of breast cancer in only 6.4%. Nonetheless, 182 of the remaining 233 of patients (78.1%) received a biopsy (pathology diagnosis) during the second hospital visit and were consequently diagnosed with breast cancer. Of the breast cancer patients who received threefold diagnostics (59.8%), final diagnosis of breast cancer was made in 86.5% during the first hospital visit. For the benign cohort, in patients who received threefold diagnostics (7.5%), a benign diagnosis during the first hospital visit was made in 50.3%. During the first hospital visit, MRI of the breast was performed in 1.5% of patients in the benign cohort and in 2.6% of the malignant cohort. 

### 3.1. Variation between Hospitals

The percentage of patients with same-day diagnosis ranged from 44% to 75% (average 62%) between hospitals for the malignant cohort. Threefold diagnostics ranged from 52% to 67% (average 60%) and with the combination of same-day diagnosis and threefold diagnostics from 37 to 63% (average 52%) between hospitals. The average number of days between first and second hospital visit varied between 1.2 to 2.6 (mean 1.9 days), the number of days between first and last hospital visit/diagnosis from 2.4 to 6.2 (mean 4.2 days), and the maximum number of days between first hospital visit and diagnosis ranged from 28 to 84 (mean 60 days) (Table S1). The three educational hospitals (hospitals 1, 8 and 9) showed a higher number of days between first hospital visit and diagnosis compared to the peripheral (3, 4 and 6) hospitals (4.6 versus 3.9).

The hospital with the lowest percentage of same-day and threefold diagnostics (36.5%) was the same hospital with the highest number of days, and thus the longest diagnostics pathway.

### 3.2. Factors Associated with the Variation in the Diagnostic Care Pathway (Malignant Cohort Only)

For breast cancer patients the total number of diagnostic care activities was associated with the following: age at diagnosis (IRR 0.998; *p* = 0.04), hospital of diagnosis (IRR range from 0.89 to 1.22; *p* < 0.0001), clinical N-stage (IRR range from 1.04 to 1.31; *p* = 0.03) and clinical M-stage (IRR 1.21; *p* = 0.01).

Factors associated with the number of days until diagnosis for malignant patients were as follows: hospital of diagnosis (HR range from 1.18 to 1.50; *p* < 0.0001), BIRADS score (HR 0.79 to 0.87; *p* = 0.0004), detected by screening (HR 1.126; *p* = 0.0030), clinical M-stage (HR 0.70; *p* = 0.0076), and tumor size (HR 0.84 to 1.16; *p* = 0.0070). For the complete case analysis (*n* = 2109), see Table 1. 

## 4. Discussion

The goal of this study was to outline the hospital-based diagnostic care pathway of women suspect for breast cancer and to identify factors which were associated with this diagnostic pathway for patients in The Netherlands. We uncovered that the diagnostic work-up of patients diagnosed with malignant disease demanded similar time, yet more diagnostic activities than of women with benign diagnosis. However, of patients with malignant disease who received threefold diagnostics, 87% had same-day diagnosis during their first hospital visit. Hospital differences were seen in the application of same-day diagnostics and threefold diagnostics and also in the number of days between hospital visits and diagnosis. Although the educational hospitals demonstrate more days until diagnosis, these hospitals conjointly have a larger, more complex treatment volume than the peripheral hospitals which may account for this difference.

We revealed a large difference in application of biopsies between the benign and malignant cohorts. In the benign cohort the majority of patients were diagnosed without biopsy during the first hospital visit (mammography and US, 71.8%; mammography, 67.7%; US, 75.9%) in contrast to the malignant cohort (mammography and US, 6.4%; mammography, 1.2%). This can be explained by the fact that abnormalities that appear on mammography do not always exhibit malignant ultrasonographic features, e.g., fluid-filled cysts are definitively diagnosed by ultrasound and do not require biopsy. This is in line with studies that have suggested that supplemental biopsy is no longer indicated due to the high negative predictive value of a negative mammogram and ultrasound [8,9,10,11].

In women with a BRCA1/2 or other high-risk mutation, screening with MRI instead of mammography is recommended in both international and Dutch guidelines [3,12,13]. The aim is to accurately detect invasive breast cancer at an early stage but also many benign lesions are found, which could explain the lower mean age in the benign cohort compared to the malignant cohort. Although in the malignant cohort the number of MRI’s was higher during the first visit compared the benign cohort, an overall higher proportion of MRI was observed in the benign cohort. We did not have any information on the gene status or familial pre-disposition of the patients in both the benign or malignant cohort.

In other studies, patients with malignant disease have been shown to obtain a diagnosis in less days than patients with a benign diagnosis [14,15]. The time between first presentation and final diagnosis was shorter for women diagnosed with malignancy, as these women were investigated more swiftly compared to the women with a benign diagnosis, possibly reflecting suspicion bias [16]. Additionally, often less than 20% of patients obtain a breast cancer diagnosis within one week from the first hospital visit [17,18]. However, Bulte et al. determined in 2013 and again in 2020 that accelerated biopsy processing could support a definitive diagnosis on the same day for the majority of suspected breast cancer patients [6,19]. In this study, 82% of patients receive a breast cancer diagnosis in one week or less. Despite the fact that the cohort of patients in this study was diagnosed in a more recent period, these results are still comparable to the outcomes of Bulte et al., since no major revisions to guidelines were implemented during this time.

Considering same-day diagnosis, results from this study are similar to previously reported results. Overall, in both cohorts combined, same-day diagnosis was achieved in 66.7% of patients (62% malignant cohort, 67% benign cohort). Barentsz et al. concluded same-day diagnosis including biopsy was feasible in the majority of patients and was able to provide almost 80% of patients with a final diagnosis on the same day and subsequently reduced anxiety in patients with a benign diagnosis [20]. Bulte et al. achieved same-day diagnosis in 65% of patients resulting from ultrasound-guided biopsy [6]. Moreover, increasing effort has been put into determining histological type, such as receptor status, using accelerated methods of biopsy processing and can be reliably used in same-day diagnosis, allowing clinicians to discuss treatment options in an early phase with the patient [21].

### Strengths and Limitations

Our study has some limitations. First of all, we did not have any information on the availability of the result of the screening mammogram in the hospital. Digital mammography was introduced in the national screening program between 2008 and 2011 which facilitated transfer of the screening mammogram to the hospital [22]. Re-evaluation of the screening mammogram in the hospital for referred patients will have occurred, yet a mammogram is not always retaken. We would have missed this care activity in our database if the mammogram was not retaken in the hospital.

Secondly, the total number of diagnostics performed could be underestimated due to the fact that we had to condense our data due to the high number of (repeated) diagnostics care activities and focus on the top five of combinations on the same day. Without condensation a total of 131 different combinations of care activities were found, which resulted in 27 combinations within the benign and 37 combinations within the malign cohort after condensation.

Moreover, we did not have any information on the use of digital breast tomosynthesis (DBT) or 3D US, which are diagnostics more commonly used nowadays in clinical practice. Nor did we have information on the physical examination of patients, so the term threefold diagnostics was based exclusively on mammography, US and biopsy. The data used in this analysis did not include the details on the result of the diagnostic activity or possible reasons for a repeated diagnostic activity, e.g., second opinion, inconclusive results or genetic predisposition status.

Finally, although the NCR is a cancer registry with nationwide coverage, it is not 100% complete [23]. The nationwide network and registry of histology and cytopathology (PALGA) discloses new cancer diagnosis, which are consequently registered in the NCR. However, the date of diagnosis in the NCR is based on what is registered in the corresponding hospital. Therefore, the date of diagnosis is registered according to the date of first histopathological confirmation of malignancy in most cases. If the date of first histopathological confirmation is unavailable, generally the date of first hospital admission is registered as date of diagnosis [24]. Consequently, in Figure 2 it appears as if malignant diagnosis have been made not based on histopathology, as this is caused by inaccuracies in the NCR. In reality, malignant diagnosis can only be made based on histopathology.

The major strength of this study was the use of financial data and the linkage to the NCR for patients diagnosed with breast cancer, allowing for the identification of factors influencing the diagnostic pathway. Additionally, the financial data provided us with the possibility to map the hospital-based diagnostic care pathway of patients with benign breast symptomatology. In both cohorts, the majority of patients received diagnostic care following the recommendations in the guidelines. Threefold diagnostics were the mainstay of malignant diagnosis, whereas most patients in the benign cohort received mammography and/or ultrasound first, followed by pathology. Malignant diagnosis is only received after histopathological confirmation.

## 5. Conclusions

To conclude, the diagnostic work-up of patients finally diagnosed with malignant disease is different from patients with benign lesions. Patients with a malignant diagnosis received their diagnosis less frequently during their first hospital visit and with a higher average number of delivered diagnostic activities compared to patients with benign lesions. However, the number of necessary hospital visits before a diagnosis was made was equal for patients with malignant or benign disease. We found that specifically threefold diagnostics (mammography, breast ultrasound and biopsy during the same visit) was especially effective in diagnosing malignant lesions. Moreover, considering the malignant care pathway, several factors influential of increasing the number of delivered diagnostic activities and number of days before diagnosis have been identified.

Ultimately, we believe this knowledge could potentially help further standardize the diagnostic pathway for patients suspect for breast cancer.

## Figures and Tables

**Table 1:** Influencing factors on number of diagnostic activities (Poisson regression *) and on number of days until diagnosis (Cox regression #) performed in the malignant cohort (complete case analysis *n* = 2109).

Variables		Number of Diagnostic Activities *					Number of Days Until Diagnosis #				
		IRR	95%CI		*p*	*p* Overall	HR	95%CI		*p*	*p* Overall
			Lower	Upper				Lower	Upper		
Age		**0.998**	**0.996**	**0.999**	**0.040**	0.040	0.999	0.997	1.003	0.815	0.810
Tumour type	invasive	ref				0.353	ref				0.119
	DCIS	1.053	0.944	1.175	0.353		0.878	0.746	1.034	0.249	
Detected by screening	no	ref				0.180	ref				**0.003**
	yes	0.975	0.940	1.010	0.180		**1.126**	**1.041**	**1.218**	**0.003**	
BIRADS	0/1/unknown	0.922	0.801	1.062	0.259		**0.789**	**0.657**	**0.949**	**0.012**	
	4	1	0.993	1.065	0.133		**0.874**	**0.81**	**0.942**	**0.000**	
	5	ref				0.100	ref				**0.000**
Multifocality	no	ref				0.112	ref				0.250
	yes	0.963	0.918	1.009	0.112		0.943	0.854	1.042	0.249	
Palpable	no	ref				0.052	ref				0.227
	yes	0.962	0.926	1.000	0.052		1.053	0.968	1.145	0.227	
Clinical T-stage	0	0.939	0.849	1.037	0.213		**0.845**	**0.728**	**0.979**	**0.026**	
(tumour size)	1	ref				0.194	ref				0.007
	2	0.963	0.923	1.006	0.09		**1.157**	**1.045**	**1.280**	**0.005**	
	3	0.989	0.914	1.070	0.787		1.091	0.919	1.294	0.319	
	4	0.895	0.792	1.010	0.074		1.156	0.936	1.428	0.178	
Clinical N-stage	0	ref				**0.035**	ref				0.260
	1	1.038	0.986	1.092	0.159		1.005	0.903	1.119	0.923	
	**2**	**1.309**	**1.004**	**1.707**	**0.047**		0.640	0.392	1.044	0.074	
	**3**	**1.162**	**1.004**	**1.344**	**0.044**		0.879	0.675	1.145	0.339	
Clinical M-stage	0	ref				**0.010**	ref				**0.0076**
	**1**	**1.208**	**1.046**	**1.394**	**0.010**		**0.699**	**0.537**	**0.909**	**0.008**	
Grade	low	ref				0.598	ref				0.351
	intermediate	1.015	0.977	1.054	0.451		1.060	0.974	1.154	0.175	
	high	1.031	0.986	1.078	0.175		1.006	0.913	1.101	0.900	
	unknown	1.020	0.938	1.109	0.640		0.975	0.832	1.144	0.759	
Localisation in breast	inner	ref				0.072	ref				0.804
	outer	0.977	0.933	1.024	0.335		1.037	0.944	1.139	0.450	
	**central**	**0.938**	**0.883**	**0.995**	**0.035**		1.016	0.892	1.158	0.811	
	overlapping	0.991	0.945	1.040	0.718		1.035	0.934	1.146	0.514	
Histology	ductal	ref				0.15	ref				0.129
	lobular	1.010	0.963	1.061	0.657		0.934	0.963	1.061	0.657	
	mixed	1.025	0.93	1.131	0.615		0.788	0.930	1.131	0.615	
	**other**	**0.926**	**0.864**	**0.994**	**0.033**		1.100	0.968	1.250	0.145	
Lateralisation	left	ref				0.777	ref				0.670
	right	0.996	0.967	1.026	0.777		0.986	0.926	1.051	0.67	
Neo-adjuvant treatment	no	ref				0.306	ref				0.630
	yes	0.997	0.945	1.053	0.922		0.991	0.877	1.120	0.887	
	no surgery	1.081	0.978	1.194	0.127		0.907	0.74	1.112	0.348	
Hospital	1	1.035	0.996	1.076	0.081		**1.294**	**1.176**	**1.423**	**0.000**	
	2	**1.139**	**1.085**	**1.195**	**0.000**		**1.123**	**1.085**	**1.356**	**0.001**	
	3	**1.224**	**1.164**	**1.288**	**0.000**		**1.500**	**1.338**	**1.670**	**0.000**	
	4	**0.893**	**0.844**	**0.945**	**0.000**		**1.184**	**1.035**	**1.355**	**0.014**	
	5	0.974	0.928	1.021	0.274		**1.296**	**1.185**	**1.416**	**0.000**	
	6	ref				**0.000**	ref				**0.000**

Bold numbers indicate significant variables (*p* < 0.001).

## Data Availability

The data presented in this study are available on request from the corresponding author. The data are not publicly available due to privacy reasons.

## Supplementary Materials

The following are available online at https://www.mdpi.com/article/10.3390/curroncol28060419/s1, Supplementary Table S1: Diagnostic pathways and time between visits and diagnosis per hospital.
