# Supplementary material for: Diagnostics in Patients Suspect for Breast Cancer in The Netherlands
Source: Curr Oncol. 2021 Nov 29;28(6):4998–5008. doi: 10.3390/curroncol28060419 (PMC8700505; doi:10.3390/curroncol28060419)
Supplement: Supplementary file 1 [file curroncol-28-00419-s001.zip › curroncol-1450244-supplementary.pdf]

Current Oncology

Supplementary Materials

Diagnostics in Patients Suspect for Breast Cancer in The Netherlands

Madelon M. Voets, Catharina G. M. Groothuis-Oudshoorn, Liset H. J. Veneklaas, Srirang Manohar, Mariël Brinkhuis, Jeroen Veltman, Linda de Munck, Lioe-Fee de Geus-Oei, Mireille J. M. Broeders and Sabine Siesling

Table S1. Diagnostic pathways and time between visits and diagnosis per hospital.

Hospital	No. of Patients	Same-Day Diagnosis		Triple Diagnostics		Same-Day & Triple Diagnosis		Days Between First and Second Hospital Visit	Days Between First and Last Hospital Visit/Diagnosis	Days Between First Hospital Visit and Diagnosis
	N	*N*	%	*N*	%	*N*	%	*Mean*	*Mean*	*Max*
1	384	272	70.8	245	63.8	237	61.1	1.7	4.0	70
3	311	206	66.2	207	66.6	179	57.6	1.6	4.2	51
4	121	88	72.7	73	60.3	70	57.9	1.2	2.4	28
6	217	162	74.7	136	62.7	123	56.7	2.3	5	59
8	419	305	72.8	271	64.7	262	62.5	1.9	3.5	65
9	784	343	43.8	406	51.8	286	36.5	2.6	6.2	84
Total	2,236	1,376	61.5	1,338	59.8	1,157	51.7	1.9	4.2	84

* Triple diagnostics is defined as the combination of mammography, US breast and pathology diagnosis on the same diagnostic care day.

Copyright: © 2021 by the authors. Licensee MDPI, Basel, Switzerland. This article is an open access article distributed under the terms and conditions of the Creative Commons Attribution (CC BY) license (http://creativecommons.org/licenses/by/4.0/).
